# Integrating AI with medical industry chain data: enhancing clinical nutrition research through semantic knowledge graphs

**DOI:** 10.3389/fdgth.2024.1439113

**Published:** 2024-10-03

**Authors:** Deng Chen, ChengJie Lu, HongPeng Bai, Kaijian Xia, Meilian Zheng

**Affiliations:** ^1^ School of Computer Science and Technology, Zhejiang Sci-Tech University, Hangzhou, Zhejiang, China; ^2^ College of International Business, Zhejiang Yuexiu University, Shaoxing, Zhejiang, China; ^3^ College of Intelligence and Computing, Tianjin University, Tianjin, China; ^4^ Department of Neurology, Second Affiliated Hospital of Soochow University, Suzhou, Jiangsu Province, China; ^5^ School of Management, Zhejiang University of Finance and Economics, Hangzhou, Jiangsu Province, China

**Keywords:** semantic knowledge graphs, clinical nutrition research, artificial intelligence (AI) integration, medical equipment, feature extraction

## Abstract

In clinical nutrition research, the medical industry chain generates a wealth of multidimensional spatial data across various formats, including text, images, and semi-structured tables. This data’s inherent heterogeneity and diversity present significant challenges for processing and mining, which are further compounded by the data’s diverse features, which are difficult to extract. To address these challenges, we propose an innovative integration of artificial intelligence (AI) with the medical industry chain data, focusing on constructing semantic knowledge graphs and extracting core features. These knowledge graphs are pivotal for efficiently acquiring insights from the vast and granular big data within the medical industry chain. Our study introduces the Clinical Feature Extraction Knowledge Mapping (CFEKM) model, designed to augment the attributes of medical industry chain knowledge graphs through an entity extraction method grounded in syntactic dependency rules. The CFEKM model is applied to real and large-scale datasets within the medical industry chain, demonstrating robust performance in relation extraction, data complementation, and feature extraction. It achieves superior results to several competitive baseline methods, highlighting its effectiveness in handling medical industry chain data complexities. By representing compact semantic knowledge in a structured knowledge graph, our model identifies knowledge gaps and enhances the decision-making process in clinical nutrition research.

## 1 Introduction

In the era of big data and precision medicine, integrating AI with healthcare data has become pivotal to uncovering new insights in clinical nutrition research. This data’s multidimensional nature presents opportunities and challenges for knowledge discovery, motivating the need for advanced analytical techniques such as semantic knowledge graphs (1). These highly complex and multifaceted data chains comprise natural language text, images, semi-structured tables, and other forms (2). They are employed to comprehend the interactions, relationships, and dependencies among various entities in the petition process, supply chain (3), and value chain. However, these data’s significant heterogeneity and diversity pose major challenges to computer processing. Therefore, appropriate techniques and methods must be employed to exploit their value fully.

Clinical nutrition research has traditionally relied on observational studies and experimental designs. However, with the advent of AI and machine learning, there is a paradigm shift towards data-intensive discovery, which can process vast amounts of medical industry chain data more efficiently. Meanwhile, due to technological advances, market trends, environmental factors, government regulations, consumer expectations, and other factors, the structure and features of medical industry chain data will change along with time dynamically (4). Therefore, the ability to update and express these data becomes increasingly important. Due to the complexity and uncertainty of the data, traditional processing techniques cannot provide accurate and relevant insights from medical industry chain data (5, 6). Data scientists and researchers face a major challenge in extracting valuable information, providing information for business decision-making, and understanding the dynamics and evolution of medical industry chain data (7, 8). The construction of knowledge graphs and extracting core features are the core technologies for unlocking the value of medical industry chain data (9). Knowledge graphs provide a semantic representation of data, capturing the relationships between various entities and helping to identify patterns that may not be obvious through traditional data analysis techniques (10, 11). Core feature extraction refers to identifying and selecting the most critical features that can represent or distinguish entities of interest in the data (12–14). However, the medical industry chain data’s evolution, dynamics, and variability require efficient knowledge graph construction and effective core feature extraction to reveal valuable insights. Using traditional methods may not yield the best results (15). Therefore, capturing the complexity, dynamics, and variability of medical industry chain data is necessary.

Despite AI’s potential in clinical nutrition, existing methods often fail to effectively handle the complexity and heterogeneity of medical industry chain data. This has led to a gap in the literature regarding developing robust, adaptive models capable of extracting meaningful insights from such data.

### 1.1 Challenges in medical industry chain knowledge graph construction

In the rapid development of the medical industry chain, the multiscale data in the medical industry chain present a significant characteristic of being massive, diverse, heterogeneous, and mutually inclusive in the granularity of fine and coarse (16). Such complexity, diversity, and magnitude should provide more information to users. However, due to the lack of effective knowledge mining, organization, and retrieval tools, such richness and diversity of information causes users to experience knowledge confusion (17). Users’ demand for efficient knowledge organization, rapid knowledge mining, and high-quality knowledge retrieval tools has become increasingly urgent (18). To enable multigrain, massive, sparse-related, and diverse heterogeneous data in many industries to provide high-quality knowledge services to users, we face the following challenges: (1) The scale and value of text data are massive and sparse in cyberspace, making it difficult for knowledge mining (19); (2) Data in multi granularity are diverse and heterogeneous, making it difficult for knowledge representation (20); and (3) User needs are diverse and variable, making knowledge retrieval difficult (21).

### 1.2 Research trends in medical industry chain knowledge graph construction

Advancements in knowledge graph construction and core feature extraction are set to revolutionize handling massive and complex data within the medical industry chain. Applying sophisticated methods is essential to extract valuable knowledge and insights from this data. Artificial intelligence, machine learning, and particularly deep learning have opened new avenues for addressing the complexities and dynamics inherent in medical industry chain data, as highlighted by Sharma et al. (22). Future research endeavors should delve into more sophisticated techniques, such as graph neural networks, reinforcement learning, and natural language processing, to enhance the efficacy of knowledge graph construction and core feature extraction in this domain.

In this paper, we develop knowledge graphs and extract core features from medical industry chain data, acknowledging its dynamic and evolving nature. We introduce a novel CFEKM method for constructing knowledge graphs and extracting core features from this data. Our approach utilizes a syntax and dependency rule-based entity extraction method for fine-grained data, sentence alignment, and comparison based on the constructed knowledge graph. The goal is to distill compact semantic knowledge from extensive multigrain medical industry chain data and organize it within a knowledge graph framework. Our model is designed to deliver robust results in relationship extraction, completion, and feature extraction, surpassing the performance of several popular feature extraction models when evaluated on real datasets and large-scale medical industry chain datasets. The imperative drives this research to fill existing gaps by employing an innovative strategy that harnesses the capabilities of semantic knowledge graphs, thereby advancing clinical nutrition research and facilitating more personalized and effective nutritional interventions.

### 1.3 Enhancing clinical nutrition with medical medical industry chain data

The intersection of clinical nutrition and the medical industry chain is a burgeoning frontier in healthcare. Clinical nutrition, which focuses on the relationship between food intake and health, increasingly relies on data-driven insights to personalize dietary recommendations and improve patient outcomes (23). With its vast data repository encompassing pharmaceuticals, medical devices, healthcare providers, and patient records, the medical industry chain offers a rich source for mining such insights (24). By integrating AI with the medical industry chain data, we can enhance clinical nutrition research and practice in several ways:

Personalized Nutritional Assessment: AI algorithms can analyze individual patient data from electronic health records to predict nutritional needs and tailor dietary plans, leading to more effective clinical interventions (25). Chronic Disease Management: Leveraging patterns within medical industry chain data, AI can identify at-risk populations and recommend preventive nutritional strategies, thus pivotal in managing chronic diseases (26). Health Management and Decision Support: Knowledge graphs constructed from medical industry chain data can provide clinicians with a comprehensive view of the nutritional landscape, facilitating informed decision-making for patient care (27).

Knowledge graph construction, while showing promise in various domains, is still emerging in its application to the medical industry. Medical industry data’s high heterogeneity, complexity, and dynamic nature challenge the process. Deng et al. (28) highlight these challenges and underscore the necessity for developing efficient and effective methods specifically tailored to the medical industry’s data characteristics.

This paper contributes to the field by proposing new methods for knowledge graph construction and core feature extraction from medical industry chain data. The contributions include (1) Introducing a syntax and dependency rule-based entity extraction method to refine the granularity of the data and supplement the knowledge graph with missing attributes. (2) Developing a sentence alignment and comparison method using the knowledge graph to enhance the efficiency and effectiveness of core feature extraction. (3) A comparative evaluation of the proposed method with existing state-of-the-art models on real, large-scale medical industry data, demonstrating its superior performance in F1 score and extraction efficiency.

We organize the paper into sections that describe the data and existing methods, introduce our proposed model and its optimization algorithm, present the experimental results, and summarize our research findings.

## 2 Data and evaluation indicators

### 2.1 Medical industry chain data

To verify the validity of the CFEKM model, this paper adopts the big data method to collect the node data of the medical industry chain. Based on the node data, medical industry chain mapping is constructed, and the collection process is divided into three steps. First, this paper collects big data information in three predefined medical industry chain dimensions: “inclusion, upstream and downstream.” This information includes annual reports, industry and industry research reports, and company announcements. This information lets us determine which entity names will likely be the first node and calculate the confidence level. The confidence level is calculated based on the ratio of the occurrences of a specific entity identified as “entity-downstream” in the company’s annual reports, announcements, and research reports over the past five years to the maximum value of all entities. The higher the confidence level, the higher the likelihood that the entity name will become the first node.

In the subsequent phase of our research, we systematically gather extensive data across three key dimensions—“containment,” “upstream,” and “downstream”—to identify the entities that could serve as the initial node. This process continues the method initiated in the first step. We scrutinize the entity names collected during the second step, comparing them for repetition or high similarity with those from the first step. Concurrently, we evaluate the confidence levels assigned to these names. Should the confidence level in the second step exceed that of the first, the entity in question is then identified as the second node in the chain.

Finally, this paper repeats the above steps to determine the medical industry chain’s third, fourth, fifth, and other nodes. The following shows the sources and sources of the pharmaceutical industry chain data collected in this paper. This paper selects three pharmaceutical industry chains: chemical preparations, medical devices and biomedicine. It collects the entity data of these medical industry chains listed in Table 1.

**Table 1:** Medical industry chain dataset.

Data source	Chemical agent#	Medical equipment	Biological medicine
Entity#	15,137	22,136	9,235

1. **Enterprise business scope and company profile**: The table begins with data items related to the business scope and company profiles, such as company names and business scopes. These data are sourced from the National Enterprise Credit Information Publicity System, accessible via http://www.gsxt.gov.cn.
2. **Patent information**: The second section focuses on patent information, including patent names and abstracts. Data are provided by the Patent Big Data Comprehensive Service Platform, available at http://www.soozl.cn/.
3. **Qualification certificates**: The third section pertains to qualification certificates, including certificate names and summary information. Data are sourced from the National Construction Market Supervision Public Service Platform, which can be queried at http://jzsc.mohurd.gov.cn/home.
4. **News information**: The news information section provides public information from the WeChat public platform, accessible via a link (the specific link was not fully displayed in the original text).
5. **Software copyright**: The software copyright section lists copyright names, with data provided by the China Copyright Protection Center, detailed information can be obtained at http://www.ccopyright.com.cn/.
6. **Administrative licenses**: The sixth section introduces administrative licenses, with data also sourced from the National Enterprise Credit Information Publicity System.
7. **Corporate websites**: The corporate websites section provides external purchase data and information on IT Orange listed companies, available at https://www.itjuzi.com.
8. **Periodic reports**: The final section concerns periodic reports, including quarterly and annual reported data, provided by the Shanghai Stock Exchange, detailed information can be inquired at http://www.szse.cn/disclosure/listed/fixed/index.html.

### 2.2 Test dataset

This paper outlines a three-step method for constructing entity datasets for three distinct medical industry chains without standardized test datasets. Initially, we selected artificial intelligence, institutional, and human entities as the primary categories. We randomly sampled 50 entities from each within the chemical agents, medical equipment, and biological medicine chains, totaling 150 entities. We then manually identify and compile related entities from their respective data sources, resulting in a dataset of 450 aligned entities, as detailed in Table 2.

**Table 2:** Medical industry chain test dataset.

Category	Chemical agent	Medical equipment	Biological medicine
Life cycle entities	50	50	50
Investment in hot entities	50	50	50
R&D dimensional entities	50	50	50

In the second step, we process the summary text of these entities, performing word separation and linguistic annotation, and cluster the data using the LDA algorithm. We categorize each cluster into three groups and align entities according to their data sources, calculating semantic and structural similarities to pair entities within each segment.

In the final step, we adjust model parameters to measure entity matching accuracy and assess model performance under varying noise levels. We collect entity data using life cycle, investment heat, and R&D dimension indicators for the three medical industry chains, with detailed information presented in Table 2. The selection of life cycle indicators is based on the understanding that medical industry chain nodes progress through distinct developmental stages, with only those in the start-up and growth phases being viable as target nodes. The investment fever indicator reflects the capital market’s preferences. In contrast, the R&D dimension indicator ensures the technological sophistication of the target nodes, which is crucial for promoting industrial upgrading and sustainable economic development.

### 2.3 Evaluation metrics

In the experimental phase of this research, a suite of established performance metrics was engaged to evaluate the efficacy of the Clinical Feature Extraction Knowledge Mapping (CFEKM) model. These metrics encompass recall, precision, and the F-measure, recognized as the cornerstones in information retrieval and data analytics. As defined by Powers (29), Recall quantifies a retrieval system’s capability to identify all pertinent instances within a dataset. Precision, conversely, reflects the proportion of retrieved instances that are contextually relevant. The F-Measure, introduced by Christen et al. (30), harmonizes Recall and Precision into a single metric, offering a balanced system performance assessment. Given the comprehensive treatment of these metrics in the extant literature, specifically within Powers (29), this manuscript eschews redundancy by directly referencing the seminal definitions and theoretical underpinnings provided therein.

## 3 Medical industry chain knowledge mapping construction

Medical industry chain knowledge mapping is a structured knowledge representation method that can represent the relationships among enterprises, entities, technologies, and other entities in the medical industry chain and the hierarchical structure of the medical industry chain. Knowledge mapping can better explain the operation mechanism of the medical industry chain, discover potential cooperation opportunities and risk points, and provide effective support for enterprises’ decision-making. The CFEKM model extracts the associated compact semantic knowledge from the massive granularity of the medical industry chain big data by mining it and organizing and expressing it using knowledge mapping. This model can significantly improve the speed of knowledge construction and thus better meet the enterprise’s demand for knowledge.

### 3.1 Medical industry chain data

Constructing a comprehensive knowledge graph is essential for its effective application. The process significantly influences subsequent operations. Typically, developing a knowledge graph encompasses tasks such as entity identification and relationship extraction. Additionally, the efficient organization and storage of the knowledge graph are crucial for rapid access and manipulation.

Creating an industry chain map in the medical industry is crucial for analysis and investment promotion. By illustrating the connections between the upstream and downstream nodes of the medical industry chain, this mapping provides precise guidance for enterprises to align with the appropriate industry chain nodes. Enterprises can accurately align themselves with industry chain nodes based on criteria such as their main sources of business income, thereby establishing a solid foundation for analyzing extended, complementary, and robust medical industry chains. The construction of a medical industry chain knowledge map can be divided into two phases: ontology and entity layer learning. Ontology layer learning includes term extraction, synonymy extraction, concept extraction, categorical and noncategorical relationship extraction, and axiomatic rule learning. The entity layer learning, or entity filling or ontology enrichment, involves the conventional steps of entity discovery and attribute supplementation—identifying the entity first and then adding its attributes and values. This process corresponds to the primary studies of entity matching and entity linking.

Given that data for non-listed companies are dispersed and lack structured entity data, the proposed solution consolidates most business data within the medical industry chain and the enterprise matching process. It primarily includes enterprise basic data (such as enterprise name, business scope, and enterprise introduction), patent information, qualification certificates, administrative licenses, construction qualifications, software copyrights, enterprise official websites, standard development, and other data dimensions.

However, we observe that factual knowledge acquired by identifying additional entities and their attribute relationships can broaden the knowledge graph’s scope. Despite the relative scarcity of this type of knowledge, it is distinguished by its abundance, dynamism, and extensive coverage. Nevertheless, the knowledge map constructed from structured medical industry chain data is limited in some entity categories and attributes to those listed in the table. From these categories and attributes, it is evident that a knowledge graph built solely with medical industry chain data presents the following two issues:

(1) The current medical industry chain entity knowledge map is limited in its representation of attributes. It primarily includes basic enterprise data such as names, business scopes, introductions, patent information, qualification certificates, administrative licenses, construction qualifications, software copyrights, official websites, and standard settings. However, it lacks comprehensive details on these entities’ life cycles and investment trends. (2) Furthermore, the relationships depicted between entities in the map are sparse, based solely on the hierarchical structure of the medical industry chain. This limited connectivity can restrict the scope of feasible solutions for knowledge mapping inference, thereby impacting the precision of the inference outcomes.

To remedy the problems of single entity attributes and sparse relationships between entities in the existing medical industry chain entity knowledge map, this paper first extracts the attributes and relationships of relevant entities from the life cycle. For the convenience of description, the lifecycle information is defined as follows:

The entity lifecycle class information is based on the entity lifecycle information composition, and the entity is represented as the following five-tuple baike_entity_info = (entity_name, desc _text, info_box, other_info, entity_type), where entity_name refers to the entity name. desc_text is the entity description text, info_box is the attribute information box, the List of entity-related dimensions and other dimensions of the entity description information is represented as other_info, and entity_type is the entity type information.

The situation in the above example is common in the fusion of data from three entity classes, and the problem is summarized in this paper as two problems:

(1) For one word with multiple meanings, the same word represents different entities in different entity class data sources. (2) Multiple words have one meaning, and the same entity has different names in different entity class data sources. How to address the situation of multiple meanings and multiple words is an important problem to be solved in this paper when fusing data from various entity class sources in the medical industry chain. At the same time, how to add accurate fused medical industry chain entity attribute relationship information to the constructed medical industry chain entity knowledge map is also a problem to be solved in this paper.

This paper addresses the entity alignment challenge stemming from homographs—instances where a single word has multiple meanings. The research integrates comprehensive entity description information by consolidating descriptions of the same entity from various entity class data sources. To tackle the issue of polysemy—where a single meaning is represented by multiple words—the paper proposes a method to map entity attribute information extracted from online entity class data onto the spatial entities of the medical industry chain. This mapping is achieved by aligning entities within the medical industry chain to their corresponding spatial entities, enriching the knowledge map with missing attributes and relationships.

### 3.2 Alignment of entities in the medical industry chain

**Figure 1:**
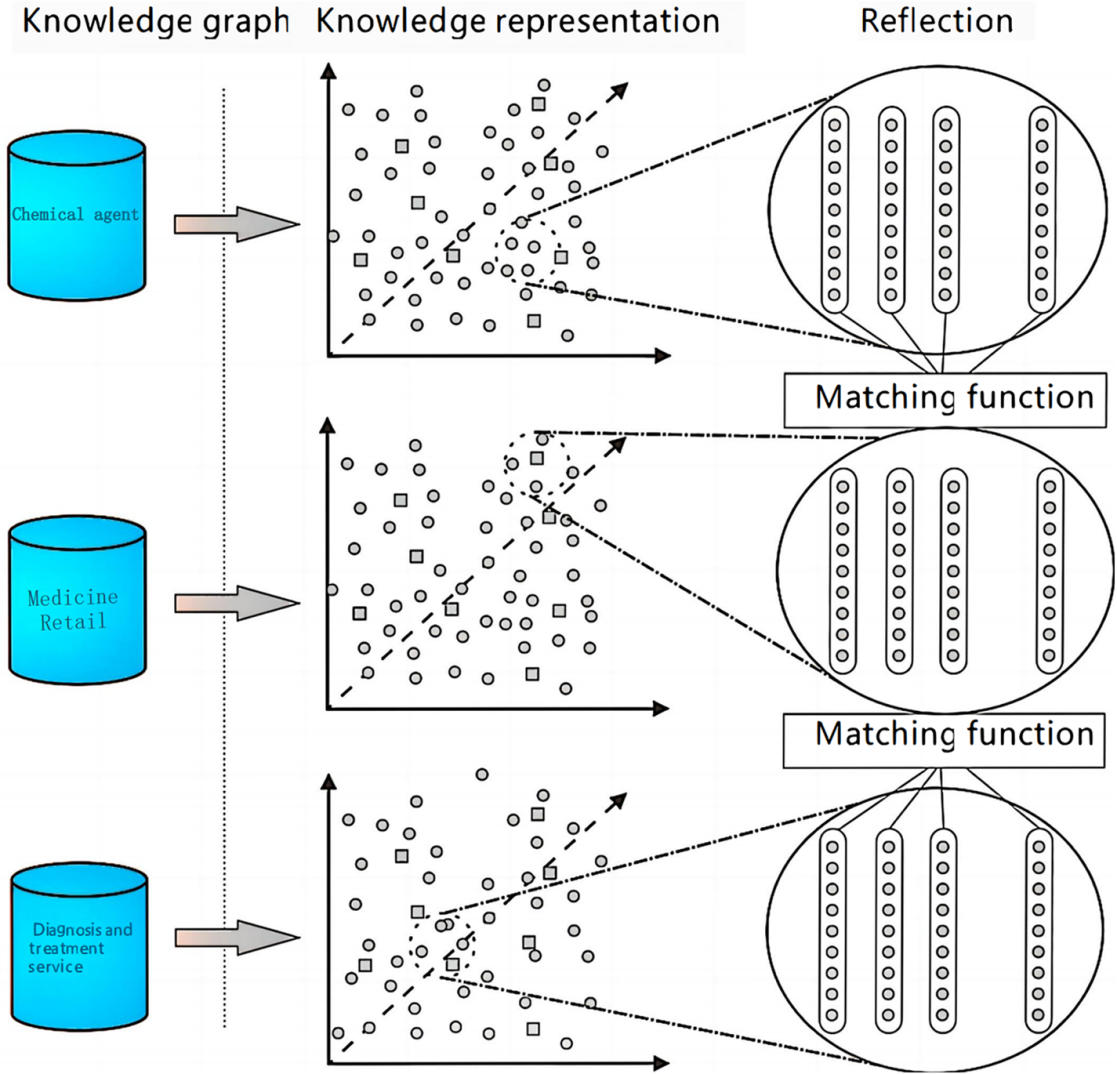
Complementary visualization of the data chunking approach for entity alignment in the CFEKM framework.

The primary objective of entity alignment is to address the challenge of linking high-quality data across diverse data sources. In this section, we employ the entity alignment technique to amalgamate entity class data from various sources, achieving a more exhaustive understanding of entity attribute relationships. To manage the extensive data volume, we implement the concept of data chunking, which simplifies computational complexity and circumvents the Cartesian entity operation inherent in entity matching. The detailed procedure is depicted in Figure 1. Initially, we combine data from three entity classes; each entity is characterized by its name and the descriptive text on the entity class web page. We then leverage the robust topical features of the Latent Dirichlet Allocation (LDA) model to cluster the entities within the database into K topics, with each entity assigned a K-dimensional topic vector. Following this, the k-means algorithm clusters the entities into manageable chunks. Ultimately, entities within each topic are categorized into three classes based on their origins, and their similarities are computed to complete the entity alignment process. Figure 1 illustrates the multi-entity alignment process.

Figure 1 illustrates the process of multi-entity alignment, highlighting the data chunking approach used to reduce computational complexity in entity matching. This method is integral to the Clinical Feature Extraction Knowledge Mapping (CFEKM) model. The figure provides a visual representation of how entities are clustered and matched, emphasizing the efficiency gains achieved by our approach.

According to the display of the entity alignment process in the above Figure 1, this paper divides the main processing process into two core parts, data chunking and entity similarity calculation, and the specific calculation steps are as follows:

#### 3.2.1 Industry standard word splitting processing

In this section, we enhance the precision of industry-standard terminology about each data dimension by undertaking a word segmentation process and conducting semantic analysis with natural language processing (NLP) techniques. We also establish synonyms and keywords for select industry-standard terms to align with various data dimensions, yielding preliminary matches between enterprises and the medical industry chain. Since entity matching across disparate data sources resembles a Cartesian entity operation, the time complexity escalates exponentially with the number of data sources involved. Such complexity becomes prohibitive when dealing with many data source entities. To mitigate this, we implement a data chunking strategy, grouping entities with identical descriptions into the same data block and calculating entity similarities, thereby reducing computational complexity.

Another benefit of using clustering for entity chunking is its ability to differentiate entities with the same name but pertain to different information classes, ensuring they are allocated to distinct data blocks. In this paper, we base our chunking approach on the desc_text field within baike_entity_info, leveraging it as a mechanism for entity disambiguation. This approach streamlines the matching process and ensures the resulting matches are more accurate and contextually relevant.

Through the above analysis, it can be seen that this paper converts the problem of performing entity chunking in the knowledge graph into the problem of clustering the desc_text text descriptions in the baike_entity_info of entities. The corpus F of the LDA used for training in this paper is the desc_text of the word entity that is a mixture of three entity classes. These data are the input because the desc_text can describe the entity well and eliminate the ambiguity between entities. The model describes the Fth entity as $\left\{V_{e1},V_{e2},\ldots ,V_{eN}\right\}$, where $V_{eN}K$ is the number of topics, i.e., the number of chunks of entities to be divided into, and the time complexity of the matching algorithm is $O\left(\prod_{i=1}^{N}|E_{i}|\right)$ If the data are chunked uniformly, the time complexity will be reduced to $O\left(\prod_{i=1}^{N}\frac{\left|E\right|}{N^{*}K}\right)$. The time complexity is effectively reduced when the value of K is larger. However, in the following, we will use the topic distribution of entity desc_text for entity similarity measurement, and the dimension of K will affect the complexity of similarity calculation. Hence, the value of K needs to be considered comprehensively.

#### 3.2.2 Industry standard word and company matching algorithm

This section elucidates our methodological strategy for leveraging word splitting and TrieTree construction to synchronize node tags and keywords within the medical industry chain. We begin by acquiring the results of word separation for node labels through a dedicated word separation interface. These results are then utilized to build a TrieTree, a benchmark for text-matching outcomes. Furthermore, we incorporate medical industry-related keywords into the dictionary for subsequent text-matching processes.

We employ the Aho-Corasick automaton algorithm to map the outcomes of node subword structures that match their respective nodes. Concurrently, the keyword node mapping table correlates the results of keyword matching to the pertinent nodes. Within the scope of this paper, we harness entity similarity to align industry standard terms with enterprises, thereby quantifying the entities’ degree of similarity. Following the initial acquisition of a candidate entity set through data chunking, we calculate entity similarity by comparing the entities’ similarities. We adopt a feature-matching approach that relies on a similarity function, which harmonizes the structural attribute similarity and semantic similarity of entity pairs. The specific entity similarity metric is delineated in Equation 1.

(1) $$\mathrm{sim}\left(e_{1},e_{2}\right)-(1-\alpha ){\mathrm{sim}}_{\text{senaulic }}\left(e_{1},e_{2}\right)+\alpha {\mathrm{sim}}_{△B}\left(e_{1},e_{2}\right)$$

where $\mathrm{sim}(e_{1},e_{2})$ is the entity-to-semantic similarity calculation function, α is the adjustment factor for both, ${\mathrm{sim}}_{\text{semantic }}(e_{1},e_{2})$ is the entity pair attribute structure similarity function, and ${\mathrm{sim}}_{△B}(e_{1},e_{2})$ is the entity pair attribute structure similarity function.

This paper considers the same entities in different medical industry chain data sources. We find that the semantic information expressed by these entities, the contextual semantic environment in which they appear, and the topics describing the text are the same. Therefore, this expressed semantic information, the contextual environment in which it occurs, and the topic describing the text are themselves another representation of the entity. This paper has trained the entity’s topic distribution matrix $\theta_{e}$ in performing data chunking. Where the entity $e_{i}$ can be expressed as $e_{i}=\{$*p* (topic $\begin{array}{ccc} & & 1\\ & & ei \end{array}),$ *p* (topic $\begin{array}{c} 2\\ ei \end{array}),\ldots ,p$ (topic $\begin{array}{c} K\\ ei \end{array})\},\sum_{k=1}^{K}p$ (topic $\begin{array}{c} k\\ e_{i} \end{array})=1$. When k=1, the model can well portray the semantic relationship of entities at the topic level.

Entities in the knowledge graph contain rich semantic relationships and are presented as graphs. Structural similarity between entities is also an indispensable factor in measuring entity similarity. Similar entities tend to have identical graph structures, e.g., they may have the same attribute values, neighboring entities, and the number of shortest paths between entity pairs in the network topology. Neighbor node counting is one of the simplest methods to calculate structural similarity. It obtains the structural similarity of an entity pair by directly counting the number of neighbors common to the set of neighbor nodes of the two entities (31). The Jaccard correlation coefficient is another simple but effective method to calculate structural similarity. It measures the similarity of an entity pair by calculating the ratio of the set of neighbors common to the concatenated set of the entity pair (32). Both methods treat neighboring attributes and relationships as having the same weight, but in practical problems, different entity attributes and relationships do not have the same magnitude of influence on entities (33). For this reason, there is an idea of assigning weights to entity attributes and relations, i.e., the calculation gives entities with more associated relations lower weights. The similarity calculation formula of this algorithm is Equation 2.

(2) $${\mathrm{sim}}_{\text{Adar }}\left(e_{i},e_{j}\right)=\frac{\sum_{NB(e)\in NB\left(e_{j}\right)\ldots B\left(e_{j}\right)}u(NB(e))}{\sum_{i}u(NB(e))}$$

where NB(e) denotes the set of relationship attributes of entity $NB(e_{i})$, and u(NB(e)) is the importance of entity e’s relationship.

The objective of this paper is to employ an evaluation system to quantify the structural similarity between entities and to refine the computation of $U_{\left(e\right)}$. Contrary to the common assumption that “an abundance of related entities results in a diminished weight for them as neighboring nodes in the calculation,” this paper posits that the significance of each attribute and entity about another can be gauged by its capacity to differentiate that entity. Accordingly, this paper adopts a method akin to TF-IDF to ascertain the distinctiveness of each attribute. The formula for this calculation is presented in Equation 3.

(3) $$u\left(NB_{i}(e)\right)=\frac{\mathrm{num}\left(NB_{i}(e)\right)}{\sum_{i=1}^{NB(e)}\mathrm{num}\left(NB_{i}(e)\right)\times \mathrm{cluster}\left(NB_{i}(e)\right)}$$

Where num $\left(NB_{i}(e)\right)$ is the number of occurrences of $NB_{i}(e)$ the number of occurrences in the knowledge graph, the more occurrences, the more important the attribute is, cluster $\left(NB_{i}(e)\right)$ is the clustering coefficient of the node. The higher the value is, the tighter the connection between the neighboring nodes of the node and the greater the possibility that its neighboring nodes belong to the same kind of entities, then the attribute belongs to the common attribute of a certain class of entities; if num (NB,(e)) is high and the clustering coefficient of the node cluster $\left(NB_{i}(e)\right)$ is very small, it means that the attribute is a common attribute of entities. Then, the differentiation of the attributes will be small.

### 3.3 Complementing entity relationships in the medical industry chain through textual data analysis

In this paper, we employ multi-granularity medical industry chain data to enhance the knowledge map, addressing the limitations of relying solely on such data for map construction. We recognize that utilizing entity class data for knowledge graph enrichment can be problematic if essential terms related to medical industry chain entities are absent or the available information is insufficient for completing entity attribute relationships. Moreover, knowledge graph inference requires substantial factual knowledge, typically found in unstructured texts like news events, seldom present in medical industry chain data and entity class web pages. We extract entity relationships from unstructured news-like texts to address these challenges and enrich our knowledge graph. However, this approach can introduce noise due to irrelevant information. Consequently, we implement a key sentence extraction algorithm to identify and extract sentences that contain more entity relations while minimizing redundancy.

This paper delves deeper into extracting entity relationships by leveraging key sentence sets. For an entity E, the key sentence set comprises the crucial and non-redundant sentences culled from the text associated with that entity. The formal representation of this set is depicted in Equation 4.

This paper further addresses entity relationship extraction employing key sentence sets. The set of key sentences of an entity E is the set of important and low redundant sentences extracted from the related text of that entity. Its formal representation is as Equation 4.

(4) $$\mathrm{sum}\left(E_{i}^{\text{sentence }}\right)=\left\{<{\mathrm{Sent}}_{i}^{\;j},\text{ weight }>|j\in [0,N]\right\}$$

Where. $<{\mathrm{Sent}}_{i}^{\;j}$, weight > is the binary representation of a sentence in the set of key sentences of the entity E, related text. Sent $t_{i}^{\;j}$ is the text content of the sentence, weight is the semantic value of the sentence at the time of sentence extraction, and N denotes the size of the set of key sentences of the entity.

In this paper, the core sentences in the corpus are extracted using the key sentence extraction algorithm to realize entity relationship extraction further. In the text, generic named entity sentences such as research reports and industry white papers contain rich entity pairs and their semantic descriptions, constituting entity relations. To accurately extract entity pairs and their semantic descriptions, this paper relies on two entities of the relationship. It defines them formally as pair $(e_{i},e_{\;j})=\{<e_{i}$, type $\left(e_{i})\right\rangle$, ¡ $e_{\;j}$, type $\left(e_{\;j})>\right\}$, where $e_{i}$ refers to an entity, type $\left(e_{\;j}\right)$ denotes the type of that entity, e.g., research report, industry white paper, etc.

The entity pair description feature sequence is a collection of terms that encapsulate the semantic interconnection between a pair of entities, potentially including their contextual information. Specifically, this paper utilizes the set of verbs found along the shortest dependency path within the dependency syntax tree of the two entities. This sequence is employed as the descriptor for the entity pair. The entity pair description feature sequence is represented as $fs(e_{i},e_{\;j})$. As shown in the definition of Equation 5.

(5) $$fs\left(e_{i},e_{\;j}\right)=\left\{w_{i}|\;\mathrm{pos}\left(w_{i}\right)\in \{v,n\},1\leq i\leq K\right\}$$

In this paper, we refine our approach to effectively mine and understand the relationships between entity pairs by employing the previously defined entity pair description feature sequences. Here, pos $\left(w_{i}\right)$ signifies the part of speech of the word $w_{i}$, where ‘v’ and ‘n’ represent verbs and nouns, respectively. The variable K denotes the length of the feature sequence. By leveraging these sequences, we aim to capture the nuanced semantic connections between entities more accurately and contribute to advancing our understanding in this domain.

Bunescu and Mooney (34) proposed and proved a hypothesis that if an entity pair $\left(e_{i},e_{\;j}\right)$ occurs in some sentence S. There exists some relationship R between the two entities in this pair $\left(e_{i},e_{\;j}\right)$, then this relationship is almost entirely concentrated in the dependent syntactic tree of the sentence S. Entities $e_{i}$ to $e_{\;j}$ on the shortest path. The discourse in question refers to the shortest dependency path between an entity pair, effectively extracting entity relations in simple sentences with only two entities. However, the complexity of Chinese sentence structures, which often include multiple components, poses a challenge for this approach. When a sentence encompasses relationships among various entities, extracting entity pairs from such a sentence necessitates incorporating grammatical knowledge, which this paper lacks.

Furthermore, in complex sentences, the shortest paths of entity pairs may overlap or intersect, complicating the extraction process for feature sequences. Typically, when a sentence includes only two entities, their relationship is more discernible, making extracting feature sequences more straightforward. The proximity of word editing distances within a sentence correlates with the strength of the semantic relationship between those words. Thus, the following guidelines should be adhered to when extracting entity pairs:

Since the dependency relationship between words decreases as the dependency distance increases, the entity pair feature sequence words should be directly connected to the entity words, i.e., words with dependency edges connected. If the sentence contains three entities $e_{1},e_{2},e_{3}$, then it can be determined that it contains two entity pairs. Pair $\left(e_{1},e_{2}\right)$, and the dependence path dependencePath $\left(e_{1},e_{3}\right)$ contains dependencePath $\left(e_{2},e_{3}\right)$, then the shortest dependence paths for each of the two entities are as Equations 6, 7.

(6) $$\begin{array}{rl} \mathrm{shortPath}\;\left(\mathrm{pair}\left(e_{2},e_{3}\right)\right) & =\text{ dependencePath }\left(e_{2},e_{3}\right)\\ \mathrm{shortPath}\;\left(\text{pair }\left(e_{1},e_{3}\right)\right) & =\text{ dependcePath }\left(e_{1},e_{3}\right) \end{array}$$

(7) $$\;\;\;\;\;\;\;\;-\mathrm{dependcePath}\;\left(e_{2},e_{3}\right)$$

This study identifies entity pairs with semantic dependencies within sentences by the semantic pointing rule. It extracts the descriptive feature sequences of these entity pairs using the feature description sequence extraction rule. Notably, when an entity pair exhibits multiple dependency paths, each triad consisting of the entity pair and the feature description sequence is considered distinct. This approach allows for recognizing various relationships between the same entity pair. The detailed mining procedure is delineated in Algorithm 1.

**Algorithm 1:** The specific mining process.

**Input:** List<cluster_no,sample_list>clusterlist=k−means(k,F) **while** *k* > *k* _*max* **do** **for** *i* ∈ (0, *k* − 1) **do** List<cluster_no,sample_list>twoCluster=2−means(F,clusterList.get(i)); **if** BIC(F, clusterList.get(i)) ¡ BIC(F, twoCluster) **then** clusterList.remove(i); clusterList.addAll(twoCluster); **end** **if** clusterList.size() == *k* **then** break; **end** **else** clusterList.remove(i); clusterList.addAll(twoCluster); **end** **if** clusterList.size() == *k* **then** break; **end** **else** *k* = clusterList.size(); **end** **end** **end**

As shown in Algorithm 1, it describes a complex data mining process encapsulated within a structured and iterative framework. It initiates with applying the k-means clustering technique to segment the dataset into k distinct clusters, each represented by a cluster number and a sample list. Subsequently, the algorithm iteratively refines these clusters by employing a two-step clustering process on the feature space F to enhance the clustering granularity. This iterative enhancement is governed by the Bayesian Information Criterion (BIC), which assesses the quality of the clustering by balancing the fit of the model to the data against the complexity of the model. Should the BIC of the refined two-cluster configuration outperform that of the original cluster, the original cluster is replaced, thereby continuously optimizing the clustering configuration. This process is reiterated until the number of clusters reaches a user-defined maximum, ensuring an adaptive and dynamic clustering solution that closely aligns with the underlying data distribution.

### 3.4 Unsupervised entity relationship extraction based on clustering

This paper examines the feature clustering of entity pairs that share the same relationship to further abstract the relationships between entities. The research is structured in a three-step process: initially, the similarity of entity pair feature sequences is assessed; subsequently, entity pairs with similar feature sequences are grouped; and ultimately, the relationship labels of entity pairs within the same cluster are extracted and appropriately assigned. Given that numerous entity pairs in the medical industry chain data exhibit identical feature sequences, the findings of this study are instrumental in enhancing the level of abstraction for entity pair relationships.

The foundation of entity clustering and classification lies in the similarity of entity-to-feature description sequences. Common methods for calculating similarity fall into two principal categories: similarity-based and distance function-based. Similarity-based methods quantify the degree of similarity between entities by computing a similarity metric. Prominent algorithms within this category include the cosine similarity calculation, the Pearson correlation coefficient, and the Jaccard similarity coefficient. Conversely, distance function-based methods represent the features of each entity as a high-dimensional vector and assess their similarity by measuring the distance between them. Notable algorithms in this category encompass the Euclidean and the Manhattan distance, among others.

Since the length of the sequence of entity pair feature descriptions extracted in this paper is not fixed, it isn’t easy to perform clustering and classification by common feature sequence similarity calculation methods. For this reason, this paper adopts the Bunescu and Mooney (34) proposed similarity calculation method based on semantic sequence kernel functions, which performs well in dealing with feature description sequences of different lengths and is widely used. The semantic relationship between entities can be portrayed by calculating the entity pair similarity using the sequence kernel function, which is calculated as Equation 8.

(8) $$K(X,Y)=\frac{1}{Z(X,Y)}\sum_{n=1}^{K}K_{n}(K,Y)$$

Where X and Y denote the sequence of feature descriptions of two entity pairs, and it is not required that the two sequences are of the same length; Z(X,Y) is the normalization factor, which is defined as Equation 9.

(9) $$Z(X,Y)=\sqrt{\sum_{n=1}^{\left|X\right|}K_{n}(X,X)\times \sum_{n=1}^{\left|Y\right|}K_{n}(Y,Y)}$$

The semantic kernel function is shown in Equation 10 and is calculated as follows:

(10) $$\begin{array}{rl} K_{n}(X,Y) & =\sum_{u\in \sum_{n}}\sum_{i\;:\;u=X[i]}\sum_{\;j\;:\;u=Y[j]}\lambda^{l(i)+l(j)}\\ & \;\times \prod_{k=1}^{n}\mathrm{SIM}\left(X_{i_{k}}\cdot \text{ word},Y_{k}\cdot \text{ word}\right) \end{array}$$

Where u represents the common subsequence of two entity pairs of feature sequences; i $=[i_{1},i_{2},\ldots ,I_{n}]$ denotes the indexed subset of the feature sequence X,X[i] is the subsequence of X. Similarly, $j=[j_{1},j_{2},\ldots ,j_{n}]$ is the indexed subset of Y;∣[i] denotes the width of X[i] in the original sequence, i.e., the difference between the maximum and the minimum value of the index; λ is the decay factor with the value range of (0,1), generally taking the value of 0.5, and its index is |[i]+|[j] indicates that the weight is inversely proportional to the sequence span, and SIM⁡() is the lexical similarity calculation function. This paper uses the entity class full-text training word vector to calculate the semantic similarity between two words by the word vector Word2Vector similarity proposed by Mikolovt et al. (35), whose calculation formula is as Equation 11.

(11) $$\mathrm{SIM}\left(W_{A},W_{B}\right)=-\frac{W_{A}\cdot W_{B}}{\left\|W_{A}\cdot \right\|W_{B}\|}$$

In this paper, the similarity between entity pairs is calculated using Equation 11, which leverages the word vectors $W_{A}$ and $W_{B}$ and their magnitudes $\left\|W_{A}\right\|$ and $\left\|W_{B}\right\|$. This method is advantageous as it accommodates feature description sequences of varying lengths and mitigates the impact of these variations on the similarity assessment. However, the primary drawback of this approach is its computational complexity, which is largely dependent on the length of the feature sequences. Fortunately, since this study utilizes the shortest dependency path as the feature description sequence, the sequence length is controlled, thereby keeping the computational expense manageable.

In the initial phase of entity pair feature sequence clustering, the paper calculates the similarity between entity pairs to evaluate their degree of resemblance. Subsequently, the paper groups entity pairs with analogous relationships into a cluster based on similarity. A similarity matrix for the entity pairs is constructed to achieve this, employing the matrix expression detailed in Equation 12, which computes the pairwise similarities. This process enables an organized and systematic approach to clustering entity pairs based on their relational similarities.

(12) $$\left(\begin{array}{ccc} k\left(1,1\right) & \cdots & k\left(1,n\right)\\ \vdots & \ddots & \vdots \\ k\left(n,n\right) & \cdots & k\left(n,n\right) \end{array}\right)$$

This paper employs the spectral clustering algorithm to cluster the feature description sequences of entity pairs, thereby categorizing the set of entity pairs into classes with high similarity. The spectral clustering algorithm relies on the similarity matrix for clustering, with *k*-means being the most widely used method. However, due to the multi-granularity nature of medical industry chain data, the exact number of entity relationships within the entity pair set cannot be predetermined, rendering the k-means clustering algorithm unsuitable.

The paper adopts the *X*-means clustering algorithm introduced by Pelleg et al. (36) to address this. This algorithm utilizes a splitting criterion to identify clustering centers and assesses the clustering outcome using the Bayesian Information Criterion (BIC) score. The *X*-means algorithm effectively addresses several limitations of the *k*-means algorithm, such as its fixed computational scale, the requirement to manually set the number of clusters X, and the propensity for the search to converge to local optima. The BIC score is calculated using Equation 13, designed to measure the model’s goodness of fit while penalizing models with larger parameters to avoid overfitting. This criterion aids in determining the optimal number of clusters by balancing the model’s fit with its complexity.

(13) $$\mathrm{BIC}\left(M_{j}\right)={\hat{l}}_{j}(D)-\frac{\;p_{j}}{2}\log|R|$$

where ${\hat{l}}_{j}(D)$ is the likelihood of model $M_{j}$ given the data $D,p_{j}$ is the number of parameters in model $M_{j}$, and |R| is the number of data points.

## 4 *CFEKM* model construction

This paper systematically constructs entity characteristics by examining three distinct medical industry chains: Chemical Agents, Medical Equipment, and Biological Medicine. The process involves a meticulous alignment of the entities within these sectors. Specifically, the paper extracts and analyzes the relationships of entities along the life cycle, investment attractiveness, and research and development (R&D) dimensions. By doing so, the study can identify and define the characteristics of entities comprehensively, taking into account the multifaceted interactions and dependencies within the Medical industry. This approach allows for a deeper understanding of the entities and their roles within the broader context of the industry, ultimately leading to a more nuanced and informed representation of their features and relationships.

### 4.1 *CFEKM* model construction

**Figure 2:**
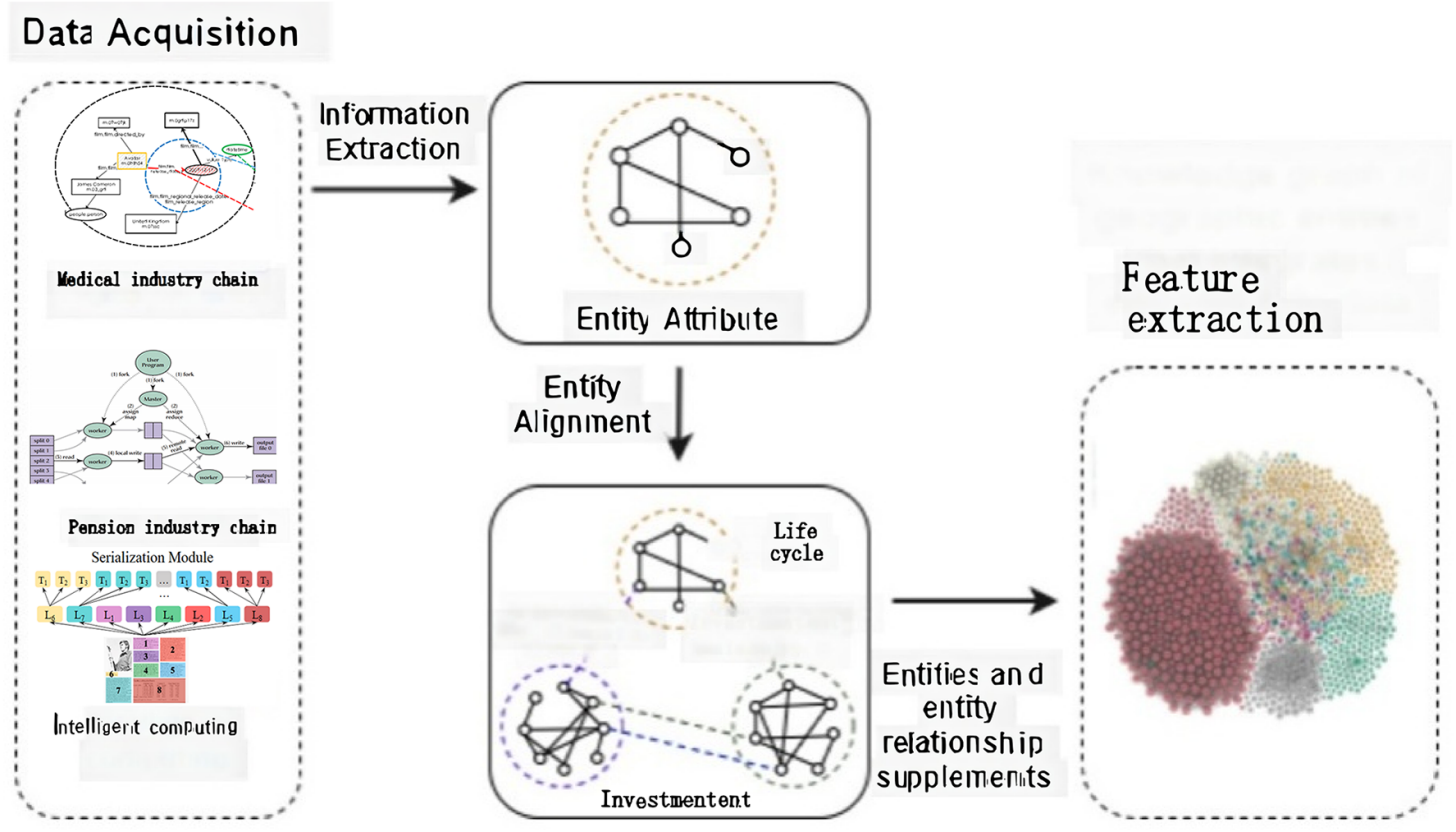
Architectural blueprint of the clinical feature extraction knowledge mapping (CFEKM) model.

This paper introduces the CFEKM (presumably a custom model name for something specific to the paper’s context) model, developed through a series of processes, including relationship label extraction, entity alignment, and feature extraction. The methodology for these extractions is outlined in Figure 2, titled “Data Acquisition,” which illustrates the specific techniques and steps employed to gather and process the necessary data for the model. This approach ensures that the CFEKM model is built upon a solid foundation of accurately identified relationships and well-aligned entities, enriched with extracted features crucial for its intended applications.

Figure 2 serves as a comprehensive illustration of the CFEKM model’s architecture, highlighting the essential elements of the model and the relationships between them. By clearly depicting the flow of information and the dynamic interactions between the various modules of the model, the diagram aids in conceptualizing the operational dynamics of the CFEKM model. It provides a clear roadmap for how data is processed, analyzed, and utilized within the model, underscoring the significance of each component in the overall system.

#### 4.1.1 Entity pairs and description of the feature extraction process

This paper delves into relationship label extraction to identify and extract suitable words from the feature description sequences of a specific class of entity pairs to serve as relationship labels for that class. The approach begins by assigning higher weights to words frequently observed within the particular category of interest. Conversely, words that exhibit high frequency across all categories have their weights diminished.

This method aims to pinpoint the most distinctive words within the category’s feature description sequences and employ these words as labels for that class. This extraction is based solely on the frequency of word occurrence, emphasizing the importance of identifying common and unique words within the category. Thus, the labels’ discriminatory power for classifying entity relationships is enhanced. This strategy is crucial for accurately categorizing and understanding the nuances of different entity relationships within the medical industry chain data.

In the initial phase of the research presented in this paper, feature terms are identified through the application of Equation 14. This equation presumably analyzes the feature description sequences and isolates the most relevant and informative terms for the subsequent study steps. By employing Equation 14, the paper aims to establish a solid foundation of feature terms that will be instrumental in relationship label extraction, entity alignment, and constructing the CFEKM model. The specific details of Equation 14 would provide the mathematical or computational basis for this feature term discovery.

(14) $$WC_{i,k}=\frac{\log_{2}\left(df_{i,k}+1\right)}{\log_{2}\left(N_{k}+1\right)}$$

In the context provided, the equation seems to be part of a process to evaluate the significance of feature terms within different categories of entity pairs. The term ${\mathrm{df}}_{i,k}$ represents the document frequency of a specific feature term i within category k, which is the count of entity pairs that include this particular feature term. On the other hand, $f_{i}$ refers to the frequency of the feature term i across all categories, which is essentially the count of all entity pairs in the dataset that contain this term. $N_{K}$ is the total number of entity pairs that belong to category k.

This approach likely aims to identify feature terms that are frequent within a specific category and relatively unique compared to their distribution across other categories. Normalizing the document frequency concerning the total number of entity pairs in the category and potentially comparing it across categories can highlight more discriminative and informative terms for classifying or analyzing entity pairs within each category. The definition formula is shown in Equation 15.

(15) $${CC}_{i}=\log\frac{N∙\max_{k\in C_{i}}\left\{WC_{i,k}\right\}}{\sum_{k=1}^{N}WC_{i,k}}∙\frac{1}{\log N}$$

where $C_{i}$ The set of all classes containing the feature word $f_{i}$ is the set of classes, and N is the total number of classes: $\frac{1}{\log N}$ One is the regular factor, which limits $CC_{i}$ the range of $[0,1]\cdot WC_{i,k}$ and $CC_{i}$ denote the importance of feature word $f_{i}$ within class k and between classes, respectively. The feature word is calculated by Equation 16, $f_{i}$ weight within the class $\left(f_{i}\right)$. Finally, the feature word with the greatest weight in each class is extracted as the relationship label of that class.

(16) $$\mathrm{Weight}\left(\;f_{i}\right)=\frac{W_{i,k}^{2}\times CC_{i}^{2}}{\sqrt{W_{i,k}^{2}\times CC_{i}^{2}}}$$

In this paper, we directly apply category labels to denote specific relationships, which is too broad to capture the nuanced semantic details of individual relationships. Words that signify the relationship between entity pairs appear within their feature description sequences. Consequently, after extracting the category labels, we employ Equation 17 to identify the word that exhibits the greatest similarity to the category label vocabulary. This approach allows us to more effectively retain the precise semantic information inherent in the relationships between entity pairs.

(17) $$\arg_{\;f_{i}}\max\mathrm{sim}\left(\;f_{i},f_{i}^{C}\right)$$

Among them. $f_{i}\in f_{s}(e_{i}.e_{\;j})$ is the tag word from that entity to pair $\left(e_{i}.e_{\;j}\right)$ from the feature sequence of that entity pair. $f_{i}^{c}$ is the category tagging vocabulary of the category in which the entity pair is located; the $\mathrm{sim}(f_{i}.f_{i}^{c}$ function calculates the similarity between the feature sequence vocabulary and the category label words.

This paper employs the semantic pointing rule to identify entity pairs with semantic dependencies within sentences. Subsequently, we extract the description feature sequences for these entity pairs using the feature description sequence extraction rule. Notably, when an entity pair exhibits multiple dependency paths, each triad consisting of the entity pair and its corresponding feature description sequence is considered a distinct entity. This approach permits multiple relationships to be associated with the same entity pair. The detailed mining procedure is outlined in Algorithm 2.

**Algorithm 2:** The specific mining process.

**Input:** $List<cluster_{n}o,sample_{l}ist>clusterList=k-means(k,F)$ **while** *k* > *k* – *max* **do** **for** *i* in (0, *k* – 1) **do** $List<cluster_{n}o,sample_{l}ist>twoCluster=2-means(F,clusterList.get(i))$; **if** BIC(F, clusterList.get(*i*)) ¡ BIC(F, twoCluster) **then** clusterList.remove(*i*); clusterList.addA11(twoCluster); **end** **if** *clusterList.size()==k* **then** break **end** **else** clusterList.remove(*i*); clusterList.addA11(twocluster); **end** **if** clusterList.size()==*k* **then** break **end** **else** *k* = clusterList.size(); **end** **end** **end**

Algorithm 2 presents a comprehensive methodology for extracting entity relationships from textual data, underpinned by a robust clustering technique. The algorithm’s operational flow begins with initializing a dataset partitioning through the *k*-means clustering approach, which segregates the data into ‘k’ distinct clusters, represented by a list of cluster numbers and their corresponding sample lists.

The algorithm then enters an iterative refinement phase, where each cluster is evaluated for potential division into two sub-clusters using a two-step clustering process. This bifurcation is contingent upon the Bayesian Information Criterion (BIC), denoted as: BIC⁡(F, clusterList.get(i)).

This BIC value measures the quality of the clustering configuration, striking a balance between the model’s fit to the data and its complexity. If the BIC of the proposed two-cluster configuration exceeds that of the existing single cluster, the original cluster is replaced, effectively enriching the granularity of the clustering.

The following conditions govern the iterative enhancement process: (1) A two-cluster configuration is proposed for each cluster ‘i’ in the range from 0 to k−1. (2) The BIC of the existing cluster is compared with that of the proposed two-cluster configuration. (3) If the BIC of the two-cluster configuration is higher (indicating a better fit with increased complexity), the original cluster is replaced by the two new clusters.

This process continues in a loop, adjusting ‘k’ dynamically based on the size of the cluster list until a user-defined maximum cluster count is reached, ensuring an adaptive clustering strategy that closely aligns with the data’s inherent structure. The inputs to Algorithm 2 include the initial number of clusters ‘k’ and the feature space ‘F’. The output is a refined list of clusters, each with an enhanced representation of the data’s underlying relationships and a deeper level of granularity.

The algorithm’s mathematical formulation is encapsulated within the BIC calculation, which is pivotal for the iterative refinement process. The BIC score is calculated using the formula:

$$\mathrm{BIC}\left(M_{j}\right)-{\hat{l}}_{j}(D)-\frac{\;p_{j}}{2}\log|R|$$

where $hatl_{j}(D)$ is the likelihood of model $M_{j}$ given the data $D,p_{j}$ is the number of parameters in model $M_{j}$, and |R| is the number of data points. This criterion is essential for the model to determine the optimal number of clusters by balancing the model’s fit with its complexity, thereby guiding the algorithm’s decision-making process in refining the clustering configuration.

#### 4.1.2 Analysis of relationship extraction results

This paper uses an entity relationship extraction method grounded in syntactic dependency rules to identify entity relationships within sentences and extract varying numbers of key sentences. The accuracy of these results is assessed through manual evaluation. The experimental findings of this paper are as follows:

1. As the number of key sentences increases, the number of extracted entity relationships increases. However, until the number of sentences is below 600, the number of extracted entity relations increases rapidly with the number of sentences. When the number of sentences exceeds 600, the number of extracted entity relations stabilizes due to the increase in redundant information in the sentences.
2. As the number of sentences increases, the accuracy of relation extraction gradually increases and eventually stabilizes. The highest accuracy rate in this paper reached 0.803.

Upon analyzing the sentences, this paper discerns that the cause for the result one is attributed to the top-ranked sentences being longer and encompassing more entity relationships. Consequently, the extraction of entity relationships accelerates with an increasing number of key sentences. Subsequently, this rate of increase stabilizes as the first approximately 600 sentences encapsulate the primary information pertinent to the entity in question. Concurrently, adding further sentences introduces redundant information, causing a decline in the quantity of extracted entity relationships.

Upon a deeper examination of the entity pair extraction results from 600 key sentences, this paper extracted 765 entity pairs. A meticulous manual review determined that our algorithm’s accuracy of entity pair extraction is remarkably high, at 0.949. After mitigating the impact of erroneous entity pairs on the results, the accuracy of relationship extraction improved to 0.935. The subsequent analysis revealed that inaccurate entity identification was the primary cause of incorrect entity pair extraction.

**Figure 3:**
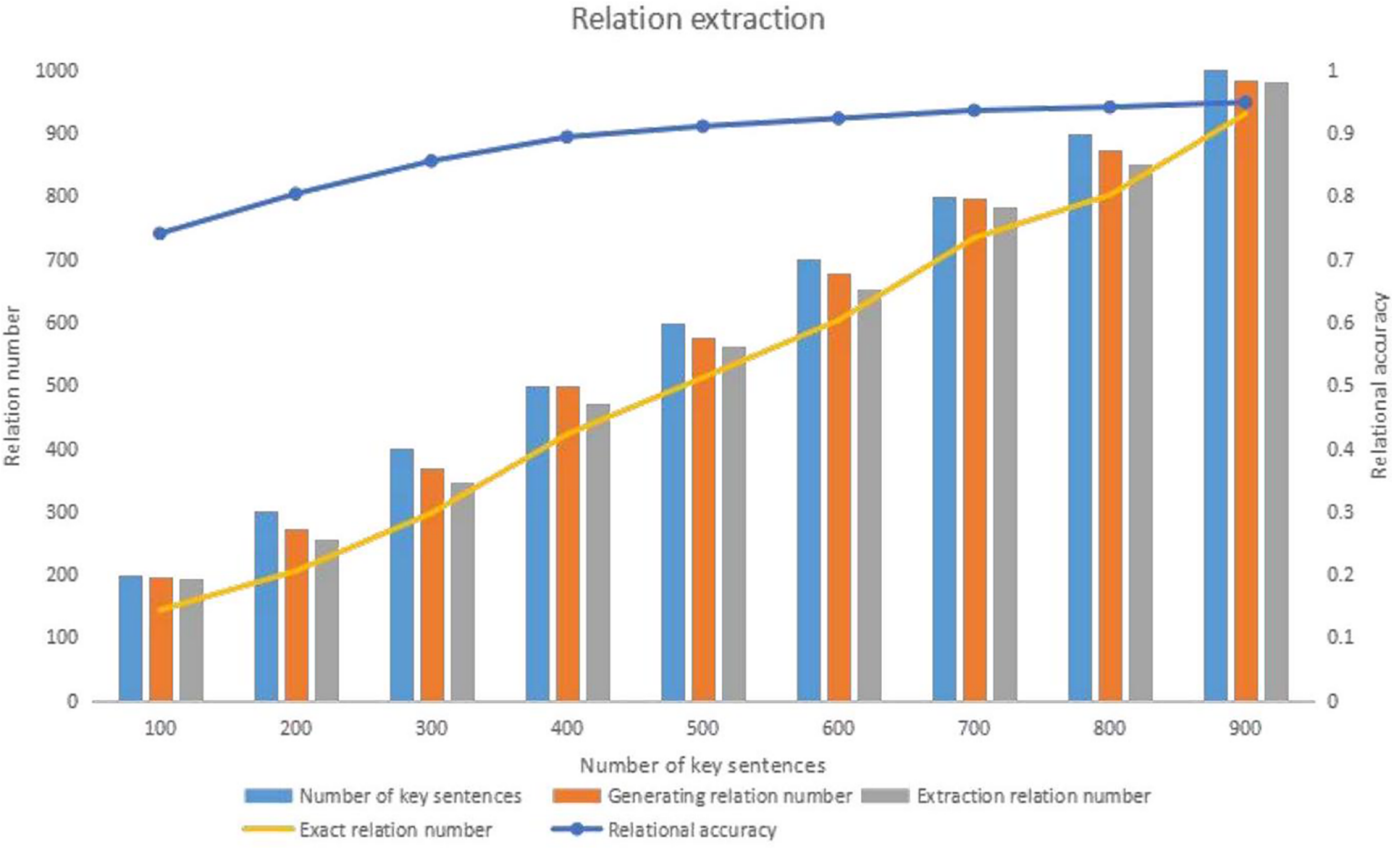
Syntactic dependency-based analysis of entity relationship extraction accuracy.

Figure 3 provides a detailed analysis of relational extraction, illustrating the impact of syntactic dependency rules on extraction accuracy. It demonstrates how the quantity of key sentences influences the extraction process and the point at which the extraction relationship stabilizes after reaching 600 sentences.

### 4.2 Entity feature extraction

The entity-relationship extraction method described earlier generates features that suffer from high dimensionality and sparsity, posing challenges for traditional classification algorithms to discern the most relevant features. To address this, our paper undertakes feature selection by leveraging deep clustering techniques, thereby diminishing the impact of extra features on developing the CFEKM model. In the course of entity relationship extraction, we encapsulate the relationships with k features, akin to the process of generating key text sentences. Additionally, to align the extracted feature set more closely with the contextual environment of the entity expansion set, we introduce dynamic features. This allows the core entity features to produce feature sets that adhere to varying contextual knowledge constraints, contingent upon the specific contextual context. As a result, the feature sets derived from entity relationship extraction exhibit enhanced informativeness and novelty, aligning more closely with practical application requirements. Utilizing the CFEKM model, we can better classify and predict entity relationships.

Given the set of related entities $\left\{e_{1},e_{2},\ldots ,e_{n}\}\subseteq \mathrm{Res}(e_{i}\right)$, we aim to extract a core set of features of length K for each entity. This task can be formulated as a 0-1 knapsack problem, leading to the following objective function:

(18) $$\begin{array}{cc} & \mathrm{maximize}\sum_{i=1}^{\left|Res(e)\right|}\sum_{\;j=i}^{\left|\mathrm{Res}(e)\right|}\sum_{a=1}^{\left|FS\left(e_{i}\right)\right|}\sum_{b=1}^{\left|FS\left(e_{\;j}\right)\right|}w_{\;f_{ei}^{a}f_{ej}^{b}}*x_{i,a}*x_{\;j,b}\\ & \text{ s.t. }\sum_{a=1}^{\left|FS\left(e_{i}\right)\right|}x_{i,a}\leq \min\left\{K,\left|FS\left(e_{i}\right)\right|\right\},x_{i,a}\in \{0,1\} \end{array}$$

In this Equation 18, K represents the predetermined size of the core feature set for each entity. The weight $w_{\;f_{ei}^{a}f_{ej}^{b}}$ corresponds to the value of selecting features $f_{ei}^{a},f_{ej}^{b}$ that contribute to the overall objective. The selection of these features is intended to fulfill the three objectives outlined in this paper. The constraints ensure that the number of features selected for each entity does not exceed the minimum of K or the total number of features available. Each feature is included (1) or excluded (0) from the core set.

(19) $$w_{\;f_{e_{i}}^{a}f_{e_{j}}^{b}}=\left\{ \begin{array}{cc} \alpha *\mathrm{rank}\left(\;f_{e_{i}}^{a}\right),\; & \text{ if }e_{i}=e_{j},a=b\;\\ -\beta^{*}\mathrm{sim}\left(\;f_{e_{i}}^{a}f_{e_{j}}^{b}\right),\; & \text{ if }e_{i}=e_{j},a\neq b\;\\ \gamma^{*}\mathrm{sim}\left(\;f_{e_{i}}^{a},f_{e_{j}}^{b}\right),\; & \text{ if }e_{i}\neq e_{j}\; \end{array}\right.$$

In Equation 19, α,β, and γ are within the range [0,1]. α and β jointly determine the level of informativeness and novelty among the features within an entity’s feature set. The parameter γ controls the extent of influence between contextual entities; a higher value of γ indicates a stronger relationship between the extracted features of different entities, meaning they are more influenced by contextual knowledge, and the opposite is also true. The feature extraction process is detailed in Algorithm 3.

**Algorithm 3:** Entity Core Feature Extraction Algorithm.

**Input:** query entity $e_{i}$, number of entity features k, number of core sentences K **Output:** entity feature set CFEKM(*e*) $List<Sentencee_{i}>\leftarrow$ Using Algorithm 1 to mine the set of key sentences with the query entity as the core; $FS(e_{i})\leftarrow$ Identify relevant entities from a collection of key sentences; **for** *i* in (0, |*FS*(*e_i_*)|) **do** **for** *j* in (0, *k*) **do** $ECF(e)=\underset{ECF(e)}{ArgMax}\sum_{i=1}^{\left|Res(e)\right|}\sum_{\;j=i}^{\left|Res(e)\right|}\sum_{a=1}^{\left|FS\left(e_{i}\right)\right|}$ $\;\;\;\;\;\sum_{b=1}^{\left|FS\left(e_{\;j}\right)\right|}w_{\;f_{e_{i}}^{a}f_{e_{\;j}}^{b}}*x_{i,a}*x_{\;j,b}$ **end** **end**

The Algorithm 3 commences its operation by identifying key sentences associated with the query entity, leveraging an existing mining process to unearth these pivotal textual data points. Subsequently, it proceeds to recognize and enumerate relevant entities from the ensemble of key sentences, setting the stage for a feature extraction endeavor. The algorithm’s essence lies in optimizing an objective function, which is meticulously formulated to maximize the informativeness and novelty of the selected features, subject to constraints that ensure the selection of a feature subset of a predetermined size. This function is elegantly crafted to balance the informativeness within an entity’s feature set and the influence of contextual knowledge from related entities. The algorithm meticulously selects features, assigning binary values to indicate inclusion or exclusion in the core feature set, thereby enabling a tailored representation of the entity that is concise and rich in pertinent information. The process is encapsulated within an iterative framework, ensuring an exhaustive exploration of the feature space and culminating in a refined set of core features succinct yet comprehensively representative of the entity’s salient attributes. The inputs to this algorithm include a query entity, the number of entity features k, and the number of core sentences K. The output is the entity feature set CFEKM(*e*), a curated collection of features that encapsulate the core attributes of the entity in question.

The CFEKM model adopts an entity extraction method based on syntactic dependency rules in response to the above-mentioned data heterogeneity and diversity issues. This method enables the model to dynamically adapt to data changes and accurately represent the evolving medical industry chain. In addition, the model effectively handles the challenges brought by large data scale and sparsity through its advanced feature extraction and alignment mechanism.

## 5 Entity alignment experiments and evaluation in multi-granularity medical industry chain data

To validate the proposed model’s effectiveness, this paper conducts experimental evaluations on a computational platform that operates Mac OS 10.14 and Ubuntu 16.04 LTS, equipped with 64 gigabytes of RAM and a terabyte of hard disk storage. The suite of tests is designed to assess the performance characteristics of the CFEKM model under the purview of a comprehensive and heterogeneous computational environment, thereby ensuring the robustness of the findings.

### 5.1 Contrast model

The Path Ranking Algorithm (PRA) (37) is a widely recognized relational path-based inference method primarily utilized in link prediction tasks within knowledge graphs. The relational paths identified by PRA are akin to Horn clauses, which allow the path-based features generated by PRA to be converted into logical rules. This conversion aids in uncovering and comprehending the latent knowledge embedded within the knowledge graph. The core principle of PRA is to forecast a specific relationship between a pair of entities by identifying a collection of relational paths that link them. The algorithm is lauded for its high interpretability and capacity to uncover inference rules autonomously.

Nevertheless, PRA is not without its limitations. First, it struggles with handling relationships that occur infrequently. Second, it is less effective in sparse data scenarios, particularly low-connected graphs. Third, extracting paths becomes computationally expensive and time-consuming in the context of extensive graphs. These drawbacks must be considered when applying PRA in various knowledge graph scenarios.

TransE, introduced by Bordes et al. (38), is a technique for gauging the plausibility of triples within vectorized knowledge graphs. Its fundamental concept is to reframe the problem of triple validity by measuring the distance between the head and tail entities. The crux of the TransE approach lies in the formulation of the scoring function, which typically leverages the inherent rationality of the relationship to project the head entity towards the tail entity, thereby assessing the entity relationship.

Drawing inspiration from the concept of word vectors, TransE allows for mapping head and tail entities in the knowledge graph into a vector space, treating their connections as relationships within a triplet. The model boasts the benefits of simplicity and rapid training times, making it an attractive choice for certain applications. However, TransE, while simple and fast, is primarily suited for one-to-one relationships and may not effectively handle the complexities of more intricate relational patterns.

### 5.2 Effect of noise on the system model

This paper deliberately introduces various noise levels to assess the system model’s resilience to noisy data effectively. To this end, datasets about Chemical Agents, Medical Equipment, and Biological medicine are created. The paper performs five experiments for each dataset to evaluate the algorithm’s performance across three key metrics: recall rate, accuracy rate, and the F. The paper provides a detailed account of the number of entities in each dataset, the number of entity groups successfully aligned by the algorithm to date, and the number of different entity types successfully matched after algorithmic alignment.

The experimental findings of this paper demonstrate that the presence of noise impacts the performance of entity alignment. However, it is observed that as the noise level in the dataset diminishes, the entity alignment performance markedly improves. Throughout the experiments, entities categorized as lifecycle entities, investment heat entities, and R&D dimension entities are matched with high precision across various datasets. The paper substantiates the effectiveness of the proposed method by comparing its performance metrics against those of other algorithms. In conclusion, the entity alignment approach introduced in this paper can effectively tackle the challenges associated with entity alignment. It exhibits a high degree of robustness to noise, along with commendable practicality and scalability. The method has also been proven to deliver high accuracy in experiments.

**Figure 4:**
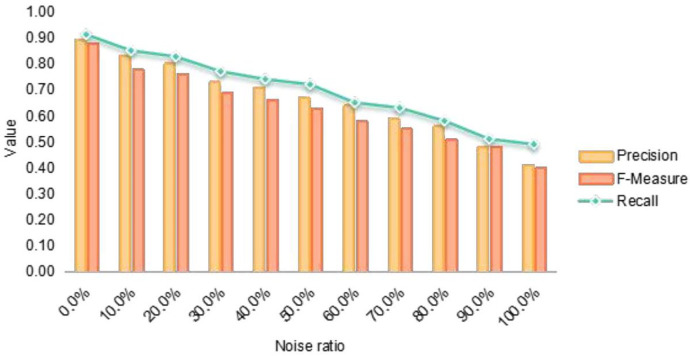
Assessment of noise impact on system model performance in medical industry chain data alignment.

As depicted in Figure 4, the successful pairing accuracy for all three types of entities declines with the introduction of varying noise levels. The table shows that the algorithm presented in this paper achieves an average accuracy of 86.4% across five experiments conducted without noise, potentially reaching as high as 91%. The experimental outcomes indicate that while an increase in noise notably diminishes the accuracy rate and F-measure, the accuracy rate remains above 50%, and the F-measure consistently exceeds 40%. It is noteworthy that although recall is typically sensitive to the addition of noise, this metric appears to have a minimal impact on the algorithm of this paper, still yielding satisfactory matching outcomes. In summary, the CFEKM method demonstrates commendable results in entity matching. Figure 4 further investigates the impact of noise on the system model’s performance, highlighting the CFEKM model’s robustness against different noise levels. The figure correlates the introduction of noise with fluctuations in recall, accuracy, and F-measure metrics, thereby emphasizing the model’s practical viability in real-world applications.

### 5.3 Entity relationship completion in the medical industry chain analysis

The integrity and precision of entity relationships within the medical industry chain are essential for a holistic representation of knowledge and for deriving subsequent data-driven clinical nutrition insights. This section focuses on completing entity relationships, highlighting their critical importance in enhancing the semantic knowledge graphs at the core of our research. The thoroughness of these relationships is not just a technical requirement but a fundamental aspect that ensures the reliability and utility of the knowledge graphs for clinical applications.

**Figure 5:**
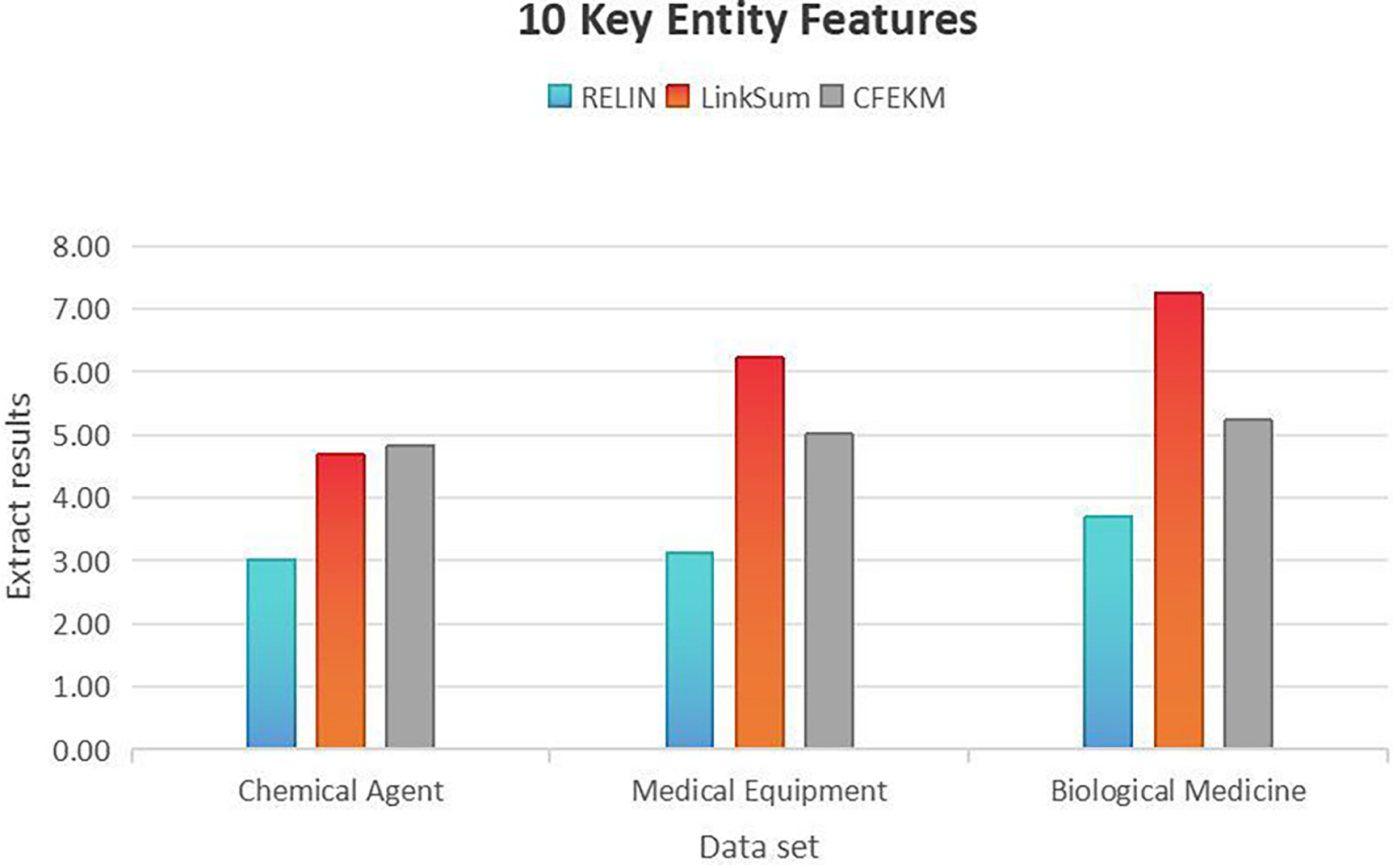
Entity relationship completion in medical industry chain knowledge graphs: CFEKM model efficacy.

The experimental outcomes from the three datasets—Chemical Agents, Medical Equipment, and smart computing—as illustrated in Figure 5, reveal that the PRA model fails to predict links, unlike the CFEKM model. Additionally, the results indicate that concentrating on the entities and their relationships along the path yields superior results compared to focusing solely on the relationships along the route. However, when comparing the performance across these three datasets, it is observed that the model presented in this paper generally excels in the context of the Biological knowledge graph. Upon further examination, this superior performance is attributed to the fact that the entities and relationships within the Biological knowledge graph are not sufficiently dense, and the Medical Equipment dataset encompasses complex relationships that challenge other models. In conclusion, the complementation method introduced in this paper demonstrates a clear advantage over other models. It is adept at handling complex knowledge graphs and can deliver satisfactory results in knowledge graph complementation. This method outperforms its counterparts and showcases its robustness and efficacy in practical applications, particularly in domains with intricate relational structures.

Figure 5 showcases the entity relationship completion within the medical industry chain, emphasizing the model’s capability to bolster semantic knowledge graphs. It compares the CFEKM model’s performance against other models, underscoring its superiority in managing complex knowledge graphs. The figure serves as a visual testament to the model’s effectiveness in enhancing the completeness and accuracy of entity relationships, which is crucial for comprehensive knowledge representation and the derivation of insightful clinical nutrition data.

### 5.4 Medical industry chain entity feature extraction

Integrating diverse data sources from the medical industry chain through knowledge fusion enriches the multi-entity relationship attributes, significantly enhancing the recall rate of the knowledge graph. However, as the graph expands, the characteristics of entities continue to increase. The medical industry chain data encompasses many entities, with sparse correlations between entity attributes, leading to high attribute dimensionality within the knowledge graph and substantial redundancy across multiple entities.

Consequently, the effective extraction of entity features from the medical industry chain data emerges as a primary methodology for assessing the model’s robustness. To rigorously validate the model, this paper extracts an ideal feature set for each dataset and conducts 100 experiments for each feature set. The outcome is determined by averaging the results of these experiments, providing a comprehensive evaluation of the model’s performance and reliability in handling complex and extensive medical industry chain data.

To validate the efficacy of the proposed algorithm, this scholarly work employs a state-of-the-art entity summarization approach, designated as RELIN, in conjunction with the widely recognized entity extraction methodology, LinkSum. These established techniques serve as benchmarks for comparative analysis within the study (39, 40). Furthermore, the research introduces an innovative set of metrics specifically crafted to assess the performance of algorithms predicated on the CFEKM framework. The delineation of these precise evaluative criteria is as follows:

(20) $$\begin{array}{cc} \text{ Agreement } & \;=\frac{2}{n(n-1)}\sum_{i=1}^{n}\sum_{\;j=i+1}^{n}\left|ECF_{i}^{l}(e)\cap {\mathrm{ECF}}_{\;j}^{l}(e)\right|\\ \text{ Quality }(ECF(e)) & \;=\frac{1}{n}\sum_{i=1}^{n}\left|ECF(e)\cap ECF_{i}^{l}(e)\right| \end{array}$$

Equation 20 is used to assess the consistency of participants’ features of interest, where ${\mathrm{ECF}}_{i}^{I}(e)$ denotes the ideal set of features, and k denotes the feature sets provided by different participants. The closer the Agreement value is to 1, the more consistent the participants recognize the features of interest and vice versa. Equation 20 is also used to evaluate the quality of the feature sets automatically generated by the algorithm, where ECF(e) denotes the feature set generated by the algorithm. The core idea of the algorithm is to assess the quality of the feature set generated by the algorithm by calculating the average overlap between the extracted feature set and the ideal feature set provided by multiple users. Quality (*ECF* (e)) The closer the value is to k The closer the quality value is to, the better the feature set generated by the algorithm is and the better it meets the user requirements, and vice versa.

In the medical equipment dataset, the *CFEKM* algorithm’s performance is closely aligned with the RELIN method, achieving scores of 4.82, 5.01, and 5.23, paralleling the RELIN method’s 62.07% success rate. This congruence indicates the *CFEKM* algorithm’s adeptness at managing the intricacies of medical equipment data, thereby ensuring a reliable and robust feature extraction process.

A comparative analysis with the LinkSum algorithm, which recorded higher scores of 4.68, 6.22, and 7.25, reveals that the *CFEKM* algorithm, while not outperforming, is a formidable contender. The proximity of scores between *CFEKM* and LinkSum suggests that both algorithms are efficacious in feature extraction, albeit through divergent methodologies and underlying mechanisms. The *CFEKM* algorithm’s consistently high scores across various datasets and key entity features signify its versatility and reliability as a feature extraction tool in diverse medical industry applications.

The inclusion of the BINDER and GPT-NER algorithms, as delineated in (41), has been instrumental in refining the *CFEKM* algorithm’s feature extraction process, particularly in the context of the Chemical Agent medical industry chain dataset at k=20. The *CFEKM* algorithm’s scores of 10.23, 11.06, and 13.02 significantly eclipse the RELIN method’s scores of 6.85, 6.68, and 7.96, underscoring its capacity for high-precision and relevant feature extraction essential for in-depth industry analysis.

Furthermore, when juxtaposed with the LinkSum algorithm, the *CFEKM* algorithm consistently secures higher scores, suggesting a more sophisticated feature extraction methodology that could enrich data representation.

In the Biological Medicine dataset, the RELIN method attains an extraction quality of 84.06% at k=20, which, while commendable, falls short of the *CFEKM* algorithm’s 66.9% success rate in the Chemical Agent medical industry chain dataset. This comparative analysis underscores the *CFEKM* algorithm’s superior performance in specific applications.

**Figure 6:**
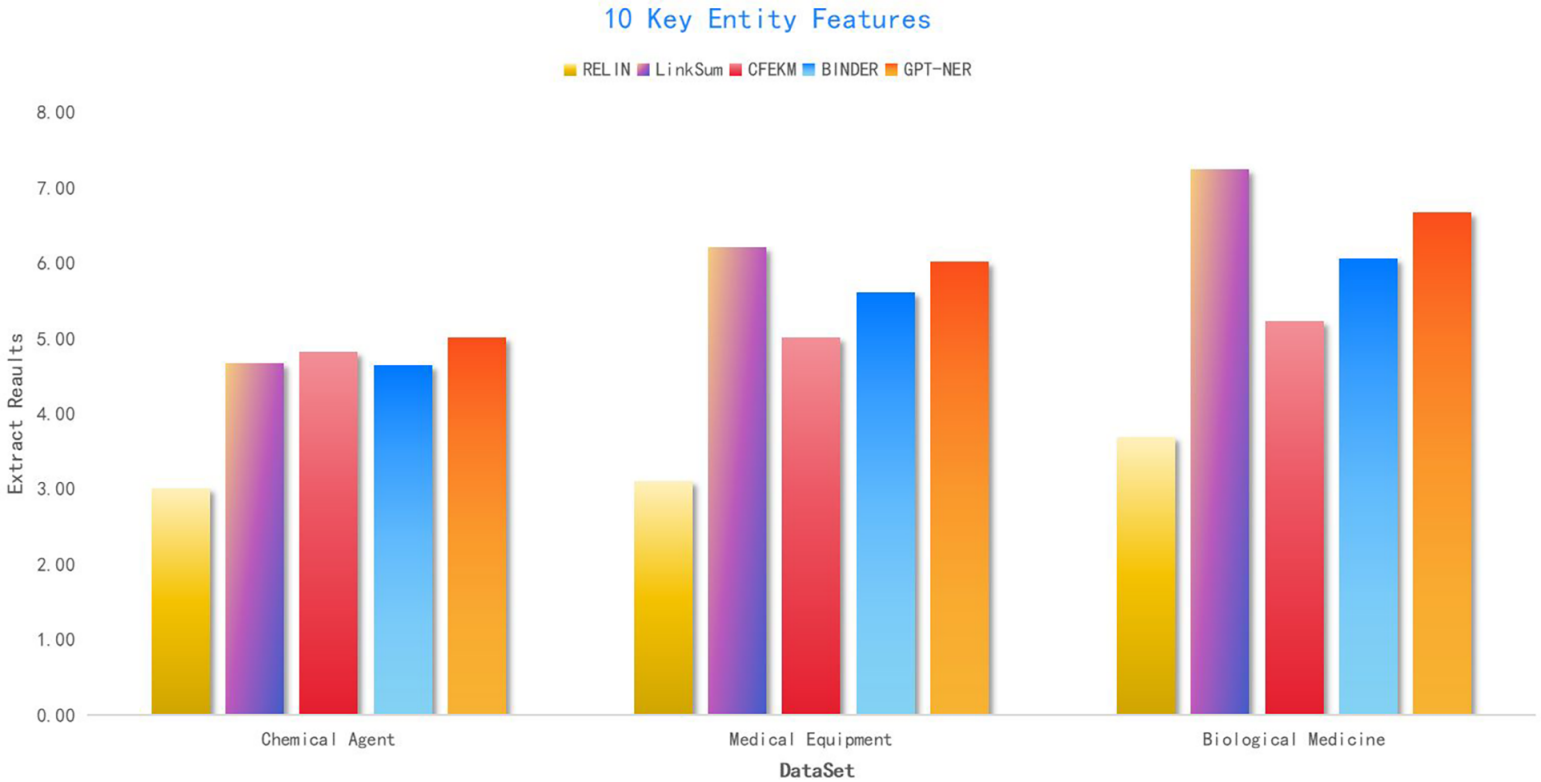
Entity feature extraction efficiency in medical industry chain datasets: a comparative study with K=10.

As shown in the Figure 6, in the Medical Equipment dataset, the RELIN method's extraction quality significantly lags, at merely 51.1% of the *CFEKM* algorithm’s success rate, thereby accentuating the latter’s effectiveness in navigating complex data relationships and ensuring a more accurate feature extraction process. In the Biological Medicine dataset, the RELIN method achieves 79.77% of the *CFEKM* algorithm’s extraction results, indicating that despite sparser inter-entity relationships, the *CFEKM* algorithm retains a competitive edge, albeit with a less pronounced performance gain.

**Figure 7:**
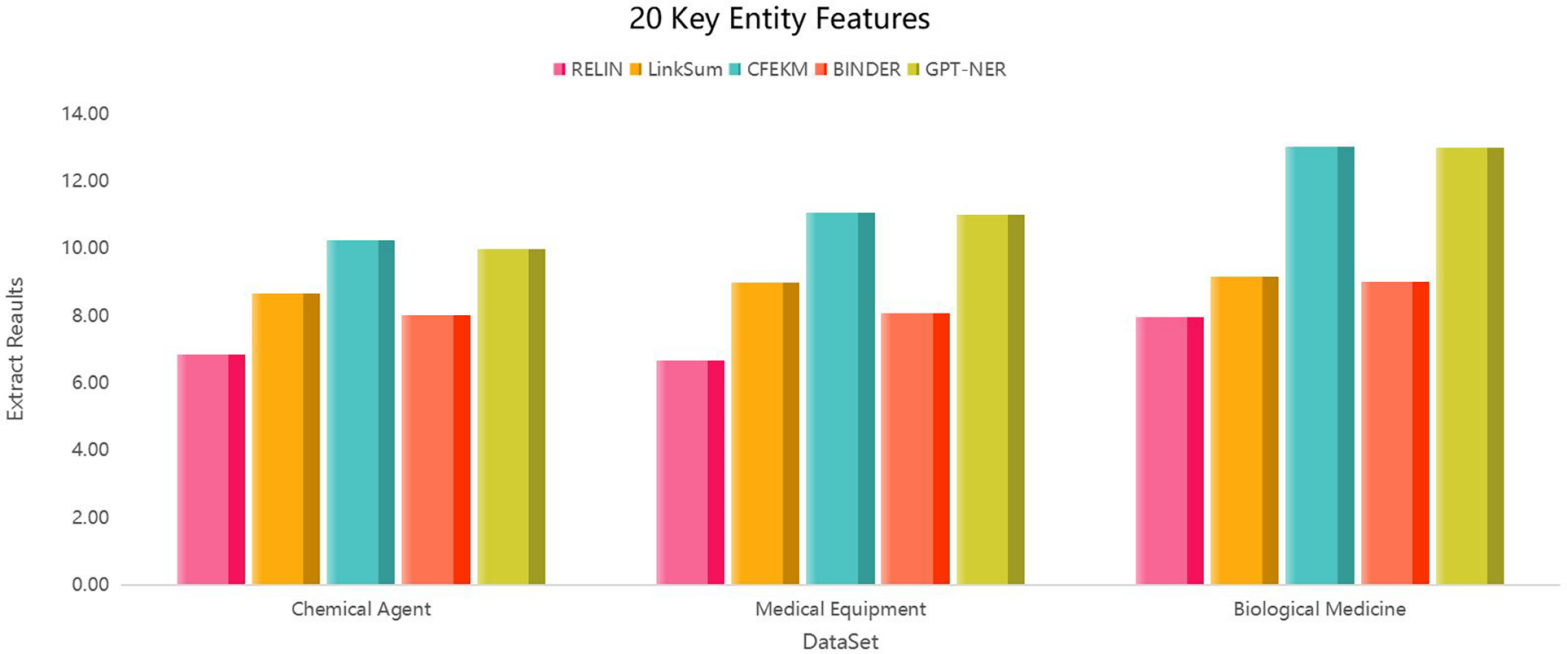
Entity feature extraction efficiency in medical industry chain datasets: a comparative study with K=20.

Figure 7 visually benchmarks the feature extraction ratio for entities within the medical industry chain, showcasing the *CFEKM* algorithm’s effectiveness in reducing dimensionality while enhancing the informativeness of the extracted features. This comparative analysis, along with Figure 7, accentuates the *CFEKM* algorithm’s potential to notably elevate the quality of feature extraction in medical industry applications.

In conclusion, the *CFEKM* algorithm, fortified with the BINDER and GPT-NER algorithms as cited in Li and Zhang (41), demonstrates a robust and versatile feature extraction capability across various datasets within the medical industry chain. Although its performance gain in the Biological Medicine dataset may not be as pronounced as in other datasets, it offers a competitive advantage, solidifying its standing as a valuable tool for feature extraction in the medical industry.

Although the model recall has decreased in some cases, this may be due to the higher complexity and noise level of certain medical industry chain data types included in the test dataset. The CFEKM model has considered these factors when designing and includes corresponding data cleaning and preprocessing steps to improve its robustness in practical applications.

## 6 Conclusion

The Clinical Feature Extraction Knowledge Mapping (CFEKM) model significantly integrates artificial intelligence with the medical industry chain, enhancing clinical nutrition research by constructing semantic knowledge graphs. The model has demonstrated robust performance in processing complex and dynamic medical data, excelling in relation extraction, data complementation, and feature extraction tasks. Future work will focus on generalizing the model for broader medical applications, ensuring scalability, customizing user experiences, integrating with additional AI techniques, processing real-time data, expanding interdisciplinary research, addressing ethical and privacy concerns, and developing an intuitive user interface. These advancements aim to solidify the CFEKM model’s position as a versatile and reliable tool in the medical industry, contributing to more personalized and effective clinical nutrition interventions.
